# Association of Southwest Meets in Galveston in Annual Session November 10 and 11

**Published:** 1914-12

**Authors:** 


ASSOCIATION OF SOUTHWEST MEETS IN GALVESTON IN ANNUAL SESSION NOVEMBER 10 AND 11.—Physicians and surgeons from Kansas, Oklahoma, Missouri and Texas—members of the Medical Association of the Southwest—met in Galveston November 10th.

At the opening of the session Tuesday morning, Dr. M. M. Graves, of Galveston, Chairman of the Committee on Arrangements, presided. He called the convention to order and stated that he was sure that the two days spent there in the discussion of scientific papers would result in untold good. He outlined the program for the entertainment of the delegates while in Galveston, and then introduced the president of the organization, Dr. S. S. Glasscock, of Kansas City, who took the chair, and the program as given in November issue of the "Red Back" was carried out.

The formation of a new medical society to be known as "The Texas Surgical Association" was definitely announced Tuesday by members of its executive committee. The organization of such an association has been simmering for the past six weeks, but assumed actual form only since the meeting of its founders in Galveston and the adoption of a constitution and by-laws.

The purpose of the association, according to Dr. J. E. Thompson, of Galveston, is to advance the interest of surgical study, research and investigation in Texas. The founders must all be surgeons, and the requirements of members who may later be added will be fixed by the committee. Dr. Thompson said that while not all the members of the executive committee are surgeons only, it was thought best to form a nucleus from which the organization may be developed. It is planned to take in 125 members at once, and to hold two meetings a year. There will be twenty-five honorary members.

A meeting of the founders will be called at Dallas on the first day of the meeting of the North Texas Medical Association in Dallas, December 9th. Members of the executive committee are: Dr. Joe Reuss, Cuero, Chairman; Dr. John T. Moore, Houston; Dr. F. Paschal, San Antonio; Dr. W. B. Thorning, Houston; Dr. Joe Becton, Greenville; Dr. J. E. Thompson, Galveston; Dr. John Smoot, Dallas.

The executive committee of the Southwestern Association is composed of Dr. M. M. Smith, Dallas; Dr. J. H. Barnes, Enid, Okla.; Dr. A. G. Dorsey, Wichita, Kan.; Dr. W. H. Stauffer, St. Louis, Mo.; Dr. E. H. Martin, Hot Springs, Ark.

The executive committee of the association in its report Wednesday recommended the adoption of the *Southwest Journal of Medicine and Surgery* as the official organ of the Association. This recommendation was adopted. The *Journal* is published at El Reno, Okla., and is edited by Dr. Fred H. Clark, Secretary of the Association. A committee consisting of Dr. E. S. Lain, Oklahoma City; Dr. Holman Taylor, Fort Worth, and Dr. C. Lester

Hall, Kansas City, was chosen to direct the publicity of the Association.

The Association also adopted a resolution authorizing the secretary to extend an invitation to the State Medical Associations of the States of Colorado, New Mexico and Louisiana to affiliate with the Association of the Southwest.

One of the most interesting addresses of the convention was the annual address of President Dr. S. S. Glasscock, of Kansas City. Among other things, the doctor said:

"We have raised the standard of our physicians and surgeons during the past few years, and can continue to do so. We should encourage universities and endowed schools with long courses for men who expect to engage in the profession of medicine, and we should urge rigid examinations.

"We can do wonders in the matter of stamping out typhoid fever and other contagious diseases. I hope to see the time come, and I believe I shall see the time, when it will be a reflection on any physician to have a case of typhoid fever or other contagious disease found in his community. You know what I mean, and you know that it should be done away with.

"There is another thing which we are practicing and which was not known a few years ago, and that is co-operation. There should be more of this. No physician or surgeon should do anything which would tend to mitigate the work of another member of the profession. There should be co-operation. We should help each other and should glory in the success of another doctor. Let's cut out that spirit of rivalry and work hard for the welfare of the profession, which means the welfare of the community. When we see that a member of the profession has gone wrong—engaged in some practices which he should not have done—let us not berate him privately or publicly, but go to him and assist him in getting right again."

The following are the officers for the ensuing year: President, Dr. J. D. Griffith, Kansas City; First Vice-President, Dr. Malvern B. Clopton, St. Louis; Secretary-Treasurer, Dr. Fred H. Clark, El Reno, Okla.

The following are the Vice-Presidents from the various States composing the organization, each State being entitled to one: Texas—Dr. A. E. Sweatland, Nacogdoches; Oklahoma—Dr. T. H. Flecher, Shawnee; Missouri—Dr. Malvern M. Clopton, St. Louis; Arkansas—Dr. F. D. Holland, Jr., Fort Smith; Kansas—Dr. Ernest F. Day, Arkansas City.

Oklahoma City was selected as the place for the next annual meeting.

The wives of the visiting physicians were entertained at a tea at Hotel Galvez on Tuesday afternoon. Wednesday's entertainment included an automobile ride for the visiting ladies in the morning, in charge of the wives of local physicians, and an oyster roast down the island in the evening, at which the Galveston Medical Association and the Galveston Commercial Association were the hosts.

